# Cases of tetanus after the Japan crisis 2011

**DOI:** 10.1186/cc10669

**Published:** 2012-03-20

**Authors:** K Morino, M Kobayashi, Y Yamada, S Yamanouchi, Y Tsujimoto, K Takeda, S Sato, S Kimura, N Mita, M Sato, S Kushimoto, S Endo

**Affiliations:** 1 Yamagata Prefectural Medical Center for Emergency, Yamagata, Japan; 2 Ishinomaki Red Cross Hospital, Ishinomaki, Japan; 3 Iwate Medical University, Morioka, Japan; 4 Tohoku University, Sendai, Japan

## Introduction

Tetanus is an infectious disease caused by tetanus neurotoxin produced by *Clostridium tetani *[1]. This bacterium resides in the soil extensively and about 100 people contract this disease annually in Japan. Tetanus is prevented by vaccines. A 1968 law required universal DPT vaccination against diphtheria, pertussis and tetanus in Japan. The survival rate with intensive care has reached more than 90% in recent years. Tetanus is said to have been found to increase in natural disasters [2]. So we will describe cases in the aftermath of the 2011 Tohoku earthquake and tsunami.

## Methods

We researched the case reports in a national database and a hospital database which could access patients' exact data. We made and analysed these case profiles.

## Results

We had nine tetanus cases in this crisis. This number was high compared with previous data. All patients lived in the Pacific coast of Tohoku districts and suffered from the tsunami. Geographically, seven patients were in Miyagi prefecture, and Iwate Prefecture had two cases. Of the nine cases, we could examine seven cases in detail. Mean age was 67 years, two were male cases and five women were injured on the day. Time to onset of symptoms such as trismus was an average of 12 days. The average was 3 days from symptom onset to medical consultation. All seven cases had some wounds, including minimal. Three had obvious wound infection. All patients had tetanus vaccine and tetanus immunoglobulin during their therapy but the time of injection was inconsistent because of the chaotic state. Four people were supported by mechanical ventilation with sedation and three out of four had tracheostomy. Three out of four with mechanical ventilation were treated with intravenous magnesium therapy to reduce spasticity. The average mechanical ventilation period was 23 days. We have no intravenous metronidazole preparation in Japan. No one had a reliable history of tetanus vaccine. No deaths were reported.

## Conclusion

We reported nine tetanus patients and investigated seven cases in detail. Older people had developed an unknown vaccination history. So we should have more opportunity to give vaccinations to older people and be careful with tetanus in disasters.
